# Inequalities in health: a comparative study between ethnic Norwegians and Pakistanis in Oslo, Norway

**DOI:** 10.1186/1475-9276-5-7

**Published:** 2006-06-29

**Authors:** Hammad Raza Syed, Odd Steffen Dalgard, Akhtar Hussain, Ingvild Dalen, Bjorgulf Claussen, Nora L Ahlberg

**Affiliations:** 1 Institute of General Practice and Community Medicine (IASAM), Department of International Community Health, Univeristy of Oslo, P.O. Box 1130 Blindern, N-0318 Oslo, Norway; 2 The Norwegian Institute of Public Health, Department of Mental Health, PO Box 4404 Nydalen, N-0403 Oslo, Norway

## Abstract

**Background:**

The objective of the study was to observe the inequality in health from the perspective of socio-economic factors in relation to ethnic Pakistanis and ethnic Norwegians in Oslo, Norway.

**Method:**

Data was collected by using an open and structured questionnaire, as a part of the Oslo Health Study 2000–2001. Accordingly 13581 ethnic Norwegians (45% of the eligible) participated as against 339 ethnic Pakistanis (38% of the eligible).

**Results:**

The ethnic Pakistanis reported a higher prevalence of poor self-rated health 54.7% as opposed to 22.1% (p < 0.001) in ethnic Norwegians, 14% vs. 2.6% (p < 0.001) in diabetes, and 22.0% vs. 9.9% (p < 0.001) in psychological distress. The socio-economic conditions were inversely related to self- rated health, diabetes and distress for the ethnic Norwegians. However, this was surprisingly not the case for the ethnic Pakistanis. Odd ratios did not interfere with the occurrence of diabetes, even after adjusting all the markers of socio-economic status in the multivariate model, while self-reported health and distress showed moderate reduction in the risk estimation.

**Conclusion:**

There is a large diversity of self-rated health, prevalence of diabetes and distress among the ethnic Pakistanis and Norwegians. Socio-economic status may partly explain the observed inequalities in health. Uncontrolled variables like genetics, lifestyle factors and psychosocial factors related to migration such as social support, community participation, discrimination, and integration may have contributed to the observed phenomenon. This may underline the importance of a multidisciplinary approach in future studies.

## Background

Health has a social gradient, i.e. people with lower socio-economic status (SES) have more health related problems than people in the other part of the SES hierarchy [1-5]. Further SE inequalities seem to increase in Western European countries [6,7]. There may be three possible pathways: increased risk of socially patterned unhealthy lifestyle in lower SES groups [8,9], unequal access to health care [10,11], and finally various exposure to material deprivation and a stressful psychosocial environment [12-16].

Inequalities in health across ethnic groups, in terms of both morbidity and mortality, have been documented in the United States [17-21] and the United Kingdom [22-27], both across social class and ethnicity. In the United States the mortality rate for black population has been at least 60 percent higher than for the white population, and trend seems to continue [28,29]. A number of studies have shown that most British ethnic minorities have higher mortality and morbidity rates compared to the majority ethnic British population [22,23,30]. The reported mortality in immigrants with South-Asian descent in the UK caused by coronary diseases, is 40 percent higher than that of Europeans [31]. This seems to be linked to the higher prevalence of diabetes in this group [32-34]. In general, mortality from both cardiovascular diseases and diabetes is more common in immigrant communities especially from South Asia [35,36] and is linked with the poor SE conditions [37-40]. Similar results have been found for South-Asians immigrants in other countries [32,41,42].

A SE gradient has been reported for health inequalities in Norway [43-47]. A study on health inequalities by SES among men has suggested an increased gradient in health inequalities for the period of 1980 to 1990. The inequality pattern appeared to be closer to the average in other European countries [48]. Similarly Norwegian women has shown SE gradient in self-reported health [49].

Inequalities in health among different ethnic groups in Norway are not extensively studied, despite the fact that earlier surveys and official reports had indicated a different morbidity pattern for immigrants in Norway compared to the native population [50]. Further, it has been reported that immigrants from non-Western countries perceive their health as poor and have high morbidity due to psychological distress and diabetes [51-53].

Self-rated health (SRH) is an important element of the clinical investigation and public health surveillance [54,55]. It is a subjective appraisal of health and a powerful predictor of survival, functional decline, future morbidity, and subsequent health service utilization. It's validity as a measure of health outcome has been explored after controlling for a variety of physical, socio-demographic, and psychosocial health indices [56]. Moreover, its importance in predicting mortality across SE categories even after adjusting for objective disease, has been reported in earlier studies [57,58]. For that reason, it has been widely used as a health outcome measure in studies investigating SE inequalities in health. Further it has been reported that immigrants in comparison to the native populations in European context often rate their health as poor [59].

Psychological distress is a measure of mental health, represented by symptoms of anxiety, depression and somatization. Studies from the Western countries have shown a social gradient; higher SES is associated with lower rate of psychological distress. Moreover, it has been suggested that differences in SES may explain differences in psychological distress between certain groups of immigrants and the native population in Western countries [60].

Self-reported diabetes was another outcome variable included in the study. The association of many vascular diseases and their risk factors with SES has been well described [61,62]. Certain risk factors implicated in the development of diabetes are also known to be associated with SES. Obesity, low physical activity, smoking, and low birth weight have all been described as risk factors for type 2 diabetes. In Western societies these factors are associated with lower SES. Thus an inverse relationship would be expected between the prevalence of type 2 diabetes and SES [63]. A few ecological studies have also described, an inverse relation between incidence of type 2 diabetes and relative affluence of the towns [63,64]. In addition to this knowledge, we also know that immigrants from South-Asia have higher prevalence of diabetes type 2 and coronary heart disease than ethnic Europeans [32,41]. This high prevalence has also been reported for the immigrants from South-Asia in Norway [52,65].

Because of the already reported poor SRH, high rates of psychological distress and diabetes among non-Western immigrants in Norway, and additionally their possible relation to SES, the aim of the present study is to investigate to which extent differences in these measures of health between Pakistani immigrants in Oslo may be explained by differences in SES.

In Norway, immigrants with Pakistani background constitute the largest non-Western immigrant group. To the best of our knowledge no previous study has particularly explained inequality in health for this ethnic group from the vantage of SE gradient. Therefore, the objective of our study is to investigate the health inequalities with respect to the selected indicators from the vantage of SE factors between ethnic Pakistanis and Norwegians in Oslo, Norway.

## Materials and methods

### Research design

This is a cross-sectional, population based study conducted as a part of a general health survey known as the Oslo Health Study. This survey was jointly organized during 2000–2001 by the University of Oslo, National Health Screening Services of Norway (now the Norwegian Institute of Public Health), and Oslo Municipality.

### Sample

**Figure 1:**
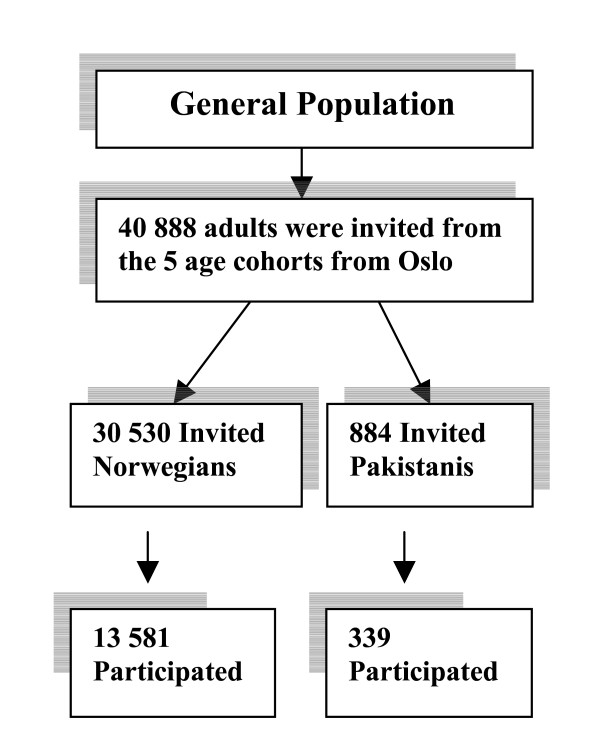
Flow chart showing study sample.

The study population included all the inhabitants of Oslo born in 1970, 1960, 1955, 1940/41 and 1924/25 (Figure 1). The Norwegian Registry of Vital Statistics provided information concerning the participant's age, gender, country of birth and residential address. Ethnicity was determined on the basis of country of birth from this register. A cross check with Statistics Norway (SSB) registers confirmed that in 99.8% of the cases the country of birth was identical to their 'country of origin'[66]. The overall attendance rate for Norwegian was 45%, and for ethnic Pakistanis it was 38%. However, the weighted prevalence of self-rated health and other analyzed variables differed only slightly between attendees and the target population, and the association measures were found to be less influenced by the self selection. The details on the methodology were described elsewhere [67].

The Norwegian Data Inspectorate approved the study. The study protocol was also reviewed by the Regional Committee for Medical Research Ethics in Norway and approved.

### Questionnaire

A questionnaire was introduced in order to collect SE information including education, employment status, occupational class, and household income. Further, a self-reported health scale indicating known state of general health, diabetes and distress was used. Originally the questionnaire was in Norwegian language, but it was translated into 11 different languages including 'Urdu'. The invitation letter and information brochure was also translated into these 11 languages [68]. The participants were allowed to give their response either in Norwegian or in their own native language.

Education was reported by the respondents as number of total school years. It was converted into primary (7–9 years), middle (10–12 years) and higher education (13 +years) categories. From the information provided by SSB, respondents were categorized into two categories with respect to their civil status, i.e. married and others. The married category included all those who were either married or registered partners. Unmarried, separated/divorced/do not live together and one partner alive were placed under the category of others. At the time of survey respondents were asked about their employment, and the response was recorded against a three item categories, i.e. yes, full time, yes, part time and no. The first two categories were combined to categorize participants into employed and unemployed categories.

The reported information about the profession of the respondents was coded according to the classification suggested by Erikson-Goldthrope [69]. This provided us with seven categories of occupation classes for respondents. These categories included higher service class, lower service class, and routine non-manual, self-employed, Technician/supervisors, skilled manual workers and unskilled manual workers. The first four categories were grouped together as white collar and the other three categories were grouped as blue collar category of occupational class. The information on household income includes all sorts of income including pension or social security before tax. It was provided by the respondents in a range from none to more than 500 thousand Norwegian Kroner (NOK). This income was categorized into three levels as low (no income to 150 thousand NOK), medium (150 to 200 thousand NOK), and high income (>200 NOK).

Self-rated health (SRH) was recorded against a four items response (bad, not very good, good, and very good), and it was converted into two categories as poor or good. Known state of diabetes was registered from the self-reported health form. The respondents were not asked to report the type of diabetes.

Distress level was measured by HSCL-10 as they reported their response against the 10 items included in the instrument. Each item was rated on a scale of 1 (not at all) to 4 (extreme) and a mean score was used as a measure of general psychological distress in the subsequent analyses. For respondents who answered at least 5 items included in the HSCL-10, data was computed by using the mean value of the remaining items reported by the respondent. A mean value of 1.85 or more was used as a marker of psychological distress [70].

### Statistical analysis

Data was analyzed using SPSS package, version 11.0. For categorical variables we used Chi-squared tests to assess the differences in distribution between groups. For continuous variables, mean and ± 2SD were provided and t-test were performed to assess the differences. All the p values reported were two-tailed. Statistical significance was set at 0.05. Both crude and adjusted prevalence of self-reported health with 95% confidence interval was reported across the socio-economic indicators. Logistic regression models were used to control for the potential confounders. The results are presented as odds ratio (OR) to indicate risk with a 95% confidence interval (CI).

## Results

The participants with ethnic Pakistani background (mean age 42.1 ± 10.8) were significantly younger (p < 0.001) than ethnic Norwegians, (mean age 50.1 ± 16.1). More than 50% of the Pakistani participants were in the middle age (i.e. 40–45 years) while Norwegians were more evenly distributed across all the age groups. All other indices of socio demographic and economic characteristics, like gender, marital status, and education, level of employment, occupational class and household income differed significantly among the participants from these two different ethnic communities (Table 1).

**Table 1:** Socio-demographic characteristics of the sample population by ethnicity (percentages)

**Variables**	**Pakistani**	**Norwegian**	**Total**	**p value†**
**Age groups(n)**	*339*	*13581*	*13920*	p < 0.0001
30 years	28.3	23.0	23.1	
40/45 years	54.0	33.8	34.3	
59/60 years	15.6	23.9	23.7	
75/76 years	2.1	19.3	18.9	
**Gender(n)**	*339*	*13581*	*13920*	p = 0.017
Male	51.3	44.8	45.0	
Female	48.7	55.2	55.0	
**Marital status(n)**	*339*	*13581*	*13920*	p < 0.0001
Married‡	90.6	47.3	48.4	
Others*	9.4	52.7	51.6	
**Education level(n)**	*339*	*13581*	*13920*	p < 0.001
Primary/basic	26.8	14.0	14.3	
Middle	33.9	24.7	25.0	
High education	39.2	61.3	60.7	
**Employment(n)**	*283*	*10859*	*11142*	p < 0.001
Employed	53.4	86.6	85.8	
Unemployed	46.6	13.4	14.2	
**Occupational class(n)**	*141*	*8807*	*8948*	p < 0.001
Blue collar	53.2	12.6	13.3	
White collar	46.8	87.4	86.7	
**Household income(n)**	*121*	*10392*	*10503*	p < 0.001
Low	66.1	29.6	30.0	
Medium	26.4	31.4	31.3	
High	7.4	39.0	38.7	

‡Married category also includes registered partners,

*other category includes unmarried, divorced, separated, and widow

† chi square test comparing Pakistanis and Norwegians

The level of unemployment was four times higher among the Pakistanis compared to the Norwegians. 66% of the ethnic Pakistanis were in the low income category, and 7% were in the high income group compared to 39% in the high income category in the Norwegians. These socio-demographic differences are in accordance with the already existing statistics on Pakistani immigrants in Norway [71,72].

Ethnic Pakistanis reported higher prevalence of poor SRH, diabetes and distress compared to the Norwegian population. This difference was significant both unadjusted and adjusted for age and sex (p < 0.001) (Table 2).

**Table 2:** Unadjusted and adjusted (age/sex) prevalence of poor-self-rated health, diabetes and distress in sample by ethnicity

**Variables**	**Pakistani**	**Norwegian**
**Poor health (n)**	*180/329*	*2940/13326*
Unadjusted	54.7	22.1*
Adjusted	60.2	21.6*
**Diabetes(n)**	*45/322*	*346/13240*
Unadjusted	14.0	2.6*
Adjusted	15.1	2.6*
**Distress(n)**	*56/254*	*1290/13037*
Unadjusted	22.0	9.9*
Adjusted	22.6	9.6*

*significant difference between Pakistanis and Norwegians, as evaluated by a chi square test, p < 0.001

Poor health was more often reported by ethnic Pakistanis for each subcategory of SE parameters (Table 3). An inverse social gradient was observed for Norwegians in respect to SRH, diabetes and distress, where as no such trend was detected for the Pakistanis. Levels of education did not show any consistent relationship neither with SRH nor diabetes among the Pakistanis; where as an opposite trend was observed for distress. Each indicator of health such as SRH, diabetes and distress showed consistent inverse relationship with income for the Norwegians. As for Pakistanis this was weak or even not existing in case of Pakistani respondents. All differences between Norwegians and Pakistanis with respect to the effect of SES, was tested for interaction by logistic regression analysis (data not shown). The following interactions were statistically significant: ethnicity* education with respect to SRH (p = 0.001) and distress (p < 0.001), ethnicity*employment with respect to SRH (p = 0.003) and distress (p < 0.001). In other words, there was a difference in the effect of education and employment on SRH and distress for the ethnic Pakistanis and Norwegians.

**Table 3:** Age and sex adjusted prevalence and 95% confidence interval for poor self-rated health, diabetes and distress in sample by ethnicity across socio-economic indicators

**Variables**	**Poor self-rated health**				**Diabetes**				**Distress**			
	
	**Pakistani**		**Norwegian**		**Pakistani**		**Norwegian**		**Pakistani**		**Norwegian**	
	**%†**	**95% CI**	**%**	**95% CI**	**%**	**95% CI**	**%**	**95% CI**	**%**	**95% CI**	**%**	**95% CI**
**Education**												
Primary/basic	56.8	46.9–66.7	37.3	35.4–39.2	14.2	7.1–21.4	4.4	3.7–5.2	15.1	5.5–24.8	17.4	16.0–18.9
Middle	51.8	42.9–60.6	25.1	23.7–26.4	11.2	4.9–17.6	2.9	2.4–3.5	21.6	12.9–30.3	11.0	10.0–12.0
High	56.6	48.1–65.0	16.9	16.1–17.8	16.4	10.5–22.3	2.2	1.8–2.5	27.6	19.3–36.0	7.4	6.8–8.0
**Paid jobs**												
No	62.7	53.8–71.6	43.8	41.8–45.7	12.9	6.9–19.0	4.2	3.6–4.9	23.0	14.5–31.4	26.3	24.8–27.9
Yes	44.6	36.3–52.9	13.9	13.1–14.6	11.0	5.6–16.4	1.2	0.9–1.5	17.9	10.3–25.5	7.0	6.5–7.7
**Occupational Class**												
Blue collar	59.6	47.9–71.3	28.7	26.5–30.9	15.6	7.1–24.1	2.0	1.2–2.7	18.4	8.0–28.8	13.6	11.8–15.3
White Collar	42.7	30.5–55.0	15.0	14.2–15.9	13.6	5.2–22.1	1.5	1.2–1.7	20.7	10.1–31.3	7.9	7.2–8.5
**Household income**												
Low	56.0	44.9–67.1	28.2	26.8–29.6	19.2	11.6–26.8	2.7	2.1–3.2	25.0	14.0–36.0	14.6	13.5–15.6
Medium	57.6	39.7–75.5	18.7	17.4–20.0	14.4	2.2–26.6	1.9	1.4–2.4	25.3	8.8–41.9	8.4	7.5–9.4
High	39.7	7.5–71.9	12.7	11.5–13.9	17.5	0–39.2	1.8	1.3–2.2	14.3	0–44.8	4.8	3.9–5.7

SE conditions, in addition to age and sex modified the odd ratios for SRH and distress but not for diabetes among the ethnic Pakistanis in the multivariate regression model (Table 4). Following the different models employed in the regression analysis it is apparent that employment status appeared to have the strongest impact on SRH and distress and to a lesser degree on diabetes.

**Table 4:** Odds ratios (95% confidence interval) of poor self-rated health, diabetes and distress for ethnic Pakistanis after inclusion of explanatory variables one by one to the adjusted model for age/sex, Norwegians as reference

**Variables**	**Models**					
	
	**Model 1**	**Model 2**	**Model 3**	**Model 4**	**Model 5**	**Model 6**
	
	**OR (95%CI)**	**OR (95%CI)**	**OR (95%CI)**	**OR (95%CI)**	**OR (95%CI)**	**OR (95%CI)**
**Self-rated health**	7.0 (5.6–8.8)	5.6 (4.4–7.0)	4.4 (3.3–5.8)	4.8 (3.4–7.0)	5.0 (3.5–7.4)	3.2 (1.8–5.5)
**Diabetes**	12.0 (8.4–17.3)	10.8 (7.5–15.6)	7.8 (5.0–12.1)	13.3 (7.5–23.7)	11.1 (6.2–20.0)	9.3 (3.8–22.9)
**Distress**	2.9 (2.1–3.9)	2.3 (1.7–3.1)	1.4 (1.0–2.0)	2.4 (1.5–3.8)	2.6 (1.6–4.3)	1.9 (0.97–3.8)

Model 1: Adjusted for age, sex and ethnicity

Model 2: Adjusted for age, sex, ethnicity and education

Model 3: Adjusted for age, sex, ethnicity and employment

Model 4: Adjusted for age, sex, ethnicity and occupational class

Model 5: Adjusted for age, sex, ethnicity and household income

Model 6: Adjusted for age, sex, ethnicity, education, employment status, occupational class and household income.

## Discussion

Our results confirm the overall poor health reported by the ethnic Pakistanis compared to the Norwegians, irrespective of SE status. The economical gradient is less marked for Pakistanis, and even an opposite trend was observed for distress. For example, individuals with a reported higher education and or higher household income have shown an association with good health among the Norwegians, where as inconsistent result was noted for the Pakistanis.

Possible explanations for the disparity in the observed associations of "health" with education and income for the ethnic Pakistanis and the Norwegians may entail that the ethnic Pakistanis at large belong to the low levels of education and income group. Given the small number of participants from Pakistan belonging to predominantly high education and income strata, it is possible that we do not observe any association of the social gradient with health although the relationship exists. The other possible explanation is that individuals with higher education from the ethnic Pakistani families were not successful in obtaining an employment that may correspond with their educational background. Therefore higher education for the Pakistani's did not result in improved economy and thereby health. Moreover underemployment may interfere with self esteem which may result in stress and depression with an obvious consequence on health.

Another factor that could contribute to the lack of positive association between education and health in the Pakistanis is inaccuracy in reporting education. In a recently conducted study in Oslo, the validity of self-reported education by immigrant communities has been discussed [73]. The conclusion was that in studies based on self-reporting, the tendency to over report education due to social desirability could not be ignored.

Among the SE indices, employment appears to have the maximum impact in explaining higher self reported morbidity among the Pakistanis. This may suggest that being employed, though not necessarily with a high income, has a positive impact on health. This is in accordance with numerous studies showing the negative effect of unemployment [74-78]. The observed unemployment in our study is in accordance with the already existing statistics in Norway. For Pakistanis reported employment rate is 44% compared to 69.3% for the total population. Moreover, described employment pattern for this ethnic group indicated that either they were self-employed with a considerable engagement in hotels and restaurants, or they were employed within elementary occupations [71]. In addition, it has been reported that immigrants do not perform as well on the labor market as natives with similar characteristics and a large proportion of immigrants from non-Western countries is characterized as self-employed marginalized, even when controlling for observed and unobserved individual characteristics [79].

The design of the study was cross-sectional and we know that inherent problems of a cross-sectional design is that the outcome (in this case self-reported health) and the exposure (in this case SE conditions) are collected simultaneously and thereby cannot predict the causality. Moreover, cross-sectional studies pay little attention to the information bias emerging from the dependent error, which means a possible correlation between the degree of error in measured exposure and measured outcome. Hence, it is possible that estimated associations between self-reported health and SE factors is falsely inflated or deflated in our study [80].

Self-reporting was the mode of data collection for both health and socio-demographic variables in this study. The data collected by self-reporting has often raised the concerns over its validity. However, self-reported health is widely used in European studies [81-83] and in American studies [84,85]. Self-reported health appeared to be an important independent predictor of all causes of mortality [86,87]. However, one cannot rule-out the possibility that cultural differences between ethnic groups may imply that they perceive their physical and mental health differently [88]. This could be the reason for the differences observed in the self-reported health measures across SE indicators for the ethnic Pakistanis in this study. In the context of our study, it seemed that while reporting their health ethnic Pakistanis were contextualizing their prevailing social condition in the Norwegian society. It might be due to this reason that constructs of health such as SRH and distress related to integrated personal perceptions and overlapping in their abilities to incorporate multiple domains of health such as psychosocial well-being, social functioning, and positive affects showed more obvious relation to the SES among Pakistanis. The same explanation could be used to describe the results of our study with regards to diabetes. A discrepancy in perception between reported diabetes prevalence and longstanding illness has been described for ethnic Pakistanis in the U.K. It was further reported that this group does not consider any illness as a longstanding illness unless it cause severe functional disability [89].

Another methodological challenge was related to the low participation rate for both the ethnic Pakistanis and Norwegians in our study. Low participation in epidemiological studies may threaten the validity and generalizability of the results due to the possibility of selective participation [90]. Though in our case, socio-demographic data provided by the central national register showed that the socio-demographic characteristics of participants were nearly the same as those of the non-participants, with slight deviation for ethnic minorities [67].

In our study, we used HSCL-10 as an investigating tool for psychological distress. Even though, it was translated into Urdu, it was not validated for the ethnic Pakistanis. Therefore, the results based on HSCL-10 for distress will have to be interpreted with caution.

## Conclusion

Inequalities in health between the ethnic Norwegians and Pakistanis were reduced, but not eliminated, when a number of socio-demographic factors were adjusted in a multivariate model. The reduction was strongest for SRH and psychological distress, where as it was weaker for diabetes. The reason for this difference could be that diabetes to a lesser degree than the other measures of health is influenced by the stressors related to migration. Unemployment seemed to be the most important explanatory factor for the difference in health. Given the context of a social-welfare state like Norway, public health policies should be developed to cope against the discrimination in employment for ethnic minorities with a view to promote health.

## Competing interests

The author(s) declare that they have no competing interests.

## Authors' contributions

HR conducted the basic study. OS participated and helped during the all phases of this study. AH contributed with the intellectual discussions and some inputs in draft. ID contributed with statistical help. BC read the script for expert comments. NA also contributed with academic discussions.
